# In Vitro Detection of Simulated Apical Root Perforation with Two Electronic Apex Locators

**Published:** 2010-02-20

**Authors:** Kiumars Nazari Moghaddam, Shahrzad Nazari, Leila Shakeri, Kiamars Honardar, Farshid Mirmotalebi

**Affiliations:** 1. Department of Endodontics, Dental School, Shahed University of Medical Sciences, Tehran, Iran.; 2. Department of Restorative Dentistry, Dental School, Shahed University of Medical Sciences, Tehran, Iran.

**Keywords:** Apex locator, Length determination, Root perforation

## Abstract

**INTRODUCTION:**

The aim of this study was to evaluate the accuracy of two electronic apex locators (Smarpex and NovApex) in detecting apical perforation.

**MATERIALS AND METHODS:**

After access cavity preparations, the working length was determined by the first examiner before and after perforation with a stereomicroscope by introducing a K-file size 10 into the canal and size 25 K-file up to the perforation site, respectively. The specimens were embedded in a 17-well plastic box containing alginate. The root canals were irrigated with chlorhexidine 0.2% (CHX) through a 27-gauge needle. Two examiners measured the root canal length twice and the mean value was calculated. The data were analyzed using Kolmogorov-Smirnov and ANOVA tests.

**RESULTS:**

The results obtained with each Electronic Apex Locator were compared with the corresponding control length. The statistical analysis showed reliable accuracies in detecting the perforation site for the two experimental electronic apex locators (60% and 80% for NovApex and Smarpex, respectively).

**CONCLUSION:**

Although no significant difference was shown between Smarpex and NovApex, these results suggest that electronic apex locators can effectively and reproducibly detect root canal perforations.

## INTRODUCTION

Working length measurement, length of preparation and obturation is a factor in determining success and failure. Better outcomes have been reported when the root canal obturation was 0-2 mm shorter than the radiographic apex, in contrast to overfilling [1]. Apical root perforation may be defined as “an artificial opening” inside the root, either by overzealous use of inflexible file with a cutting tip, or over enlarging curved root canal and pathologic processes such as root resorption which results in a communication between pulp space and periodontal ligament. The etiology, location, as well as the size of the perforation and the delay time before perforation repair, are significant factors in prognosis and treatment planning [2][3]. Accurate working length determination is mandatory for proper root canal preparation and intra canal medicament placement. Four methods for determining the correct working length are tactile sensation, radiography, bleeding point and electronic apex locators (EALs). In spite of its popularity, X-ray images have many disadvantages including highly technique-dependant, providing a two-dimensional representation of the three-dimensional object, i.e. providing no information of bucco-lingual aspect of root canal system [4]. The first use of EALs to measure root canal length was reported by Custer et al. [1]. The first generation of EALs was developed by Suzuki and Sunada [2]. All modern EALs are able to detect root perforations and lateral canals with a clinically acceptable limitations [5][6]. Recent studies revealed that EALs are acceptable clinical tools in detecting root perforations in the middle third of root canal [7]. In addition, determining the working length of the coronal segment of root canal in teeth with oblique horizontal root fractures is possible via EALs [8]. Any connection between the root canal and the periodontal membrane, such as root fracture, cracks and internal or external root resorption will be recognized by the EAL, which serves as an excellent diagnostic tool [9][10][11]. Recently, a new version of EALs was introduced to the market with the capability of working in bleeding and postulant fields. The aim of this study was to evaluate the accuracy of NovApex compared to the Smarpex in detecting simulated root canal perforations.

## MATERIALS AND METHODS

Twenty five human extracted mandibular molar teeth with developed apices were collected. They were stored in distilled water containing 10% formalin and kept refrigerated before use. The teeth were examined visually to detect fractures or cracks or any sign of root resorption. All calculus and residual organic debris in the outer surface of the roots were ultrasonically removed. (EMS Piezon® Master 600/Nyon, Switzerland). Digital images (DentiMax, LLC/AZ, USA) were taken to evaluate root canal anatomy, and to identify the radiographic apex and exclude teeth with calcified main canals. Exclusive criteria were root fracture, crack, root resorption and old root canal filling materials. Overall, 17 mandibular molar teeth met the inclusion criteria. The cusps were flattened with a tapered diamond bur (D&Z, Switzerland) using a high-speed handpiece under water coolant, to establish a level surface to serve as a stable, unequivocal reference platform for all measurements. Standard access cavity preparations were completed and the coronal pulp was excavated. The root canals were irrigated with Chlorhexidine 0.2% (CHX) (Darousazi-Najo Co., Tehran, Iran) using a 27- gauge needle (Supa Medical Devices Co., Tehran, Iran) and dried with cotton pellets. Canal patency was confirmed using a size 0.8 stainless steel K-file (Mani Co., Japan) inserted into the mesiobuccal root canal before instrumentation and irrigation of the canal system [8]. The actual length was determined by introducing a K-file size 10 (Mani Co., Japan) into the canal until the tip of the file became visible from the major apical foramen with a stereomicroscope (Zeiss Stemi, Carl Zeiss/GmbH, Germany) under ×15 magnification. Working length was considered 0.5 mm shorter than this length. Coronal pre-flaring of the canal was passively performed using #2 and #3 Gates Glidden drills [12][13][14]. Then apical preparation was performed with #10, #15 and #20 stainless steel K-files (Mani Co., Japan). Therefore, the size of apical constriction was set at 0.2 mm. Irrigation was provided with CHX 0.2% [15][16]. During canal instrumentation, the apical patency was maintained via insertion of a K-file size 8 through the foramen. At the end of instrumentation, a K-file size 20 was advanced into the canal until its tip was observed through the apex under stereomicroscope with ×10 of magnifications. While a K-file size 20 was held at working length into the canals, the teeth were intentionally perforated with a bur (Kave Co., Tehran, Iran) fixed in a high-speed handpiece under water coolant with 45 degrees angulation to the tooth long axis until the file was seen through the perforation surface. The perforation site was located at the buccal surface, 3 mm above the apical foramen.

K-file size 25 was used to produce an oval shape perforation emulate clinical position of the perforation in mesiobuccal root of mandibular molars. The teeth were embedded in an alginate model [13][14][15][16][17][18][19][20]. This model was made of plastic and alginate impression material (IRALGIN, Golchai Co., Tehran, Iran). The alginate was prepared according to the manufacturer’s instructions and packed into the model boxes separately. Cotton pellets were used to remove excess CHX from the pulp chamber. The electrode lip was attached to the alginate. The two EALs used were NovApex (VDW Co., Munich, Germany) and Smarpex (META BIOMED Co., Cheongju-city, Chungbuk, 361-140, Korea). They were utilized according to their manufacturers’ instructions. All measurements were performed by two examiners who were not informed of the perforation site. Each tooth was measured two times with EALs and the mean value was calculated. The readings of each examiner were recorded and the data were analyzed using SPSS (version 11.0 for Windows, SPSS Inc., Chicago, IL, USA), Kolmogorov-Smirnov (KST) and Pearson’s co-efficient of correlation test and analysis of variance (ANOVA). The P value was set at 0.05.

## RESULTS

The true canal lengths up to perforation site were determined both by observation (TP) and EALs’ measurements. Less than 30 specimens were used; therefore, KST test were carried out for evaluating the normal distribution of variables among specimen. ANOVA test showed no statistical significant difference between the experimental EALs (P=0.408). Comparison between the TP measurements and Smarpex group showed high correlation; however this was not the case for NovApex readings (Pearson’s coefficient of correlation). The average measured deviation (±SD) in relation to the apical perforation was 1.6 mm for the Smarpex and 1.3 mm for the NovApex respectively. The limit of ±0.5 mm from the apical perforation was attained by the Smarpex in 80% and by the NovApex in 67%, of all readings (Table 1).

**Table 1:** Distribution of the file tip position in relation to the root perforation

**Distance from apical perforation**	**Smarpex**		**NovApex**	
	**(n)**	**(%)**	**(n)**	**(%)**
**<-0.5 mm**	1	6.6	1	6.6
**-0.5 to 0.5 mm**	12	80	9	60
**>0.5 mm**	2	13.4	5	33.4

## DISCUSSION

Although root perforation occurs in a small percentage of root canal treatments, it is a serious procedural mishap which reduces long term prognosis. Early diagnosis and treatment of such an accident can prominently improve the outcome. A precise device which locates root perforations is essential for successful treatment [1]. Root perforations in the bucco-lingual aspect of the root are difficult to diagnose radiographically even for the well trained endodontist [5][21][22]. The use of EALs for determining the working length during endodontic therapy provides the clinician with the reliable measurements for each individual root canal[23][24]. Since their introduction, especially the most recent generation, EALs have gained popularity. According to manufacturer's claim, these EALs are able to work properly in the presence of exudate and blood, e.g. perforation cases. We applied error tolerance in our study defined as ±0.5 mm distance from apical foramen. Also, in contrast to the apical terminus, no constriction can be felt in the site of perforation during treatment. Therefore, maintaining the obturation limit, short of the external root surface, is essential and decreases the risks of overfilling [5][25]. As mentioned, the diameter of the apical foramen affected the accuracy of EALs in some studies [26], therefore the diameter of the perforations was kept as consistent as possible. According to Pecora et al., we conducted coronal preflaring before measurements in order to improve exact determination of the working lengths by apex locators [14]. Canal irrigation was performed with CHX 0.2% based on Ebrahim and Ozsezer’s studies who found accurate canal length measurement in the presence of CHX in the root canals enlarged with large-size files [15][16].

NovApex and the Smarpex detected perforations at a -0.5 to 0.5 mm distance range in 60% and 80% of measurements, respectively. As over 50% of cases are detected, this method should be used alongside other diagnostic tools.

## CONCLUSION

According to the findings of the present investigation, digital screening EALs locate perforation site more accurately compared to analogue ones, suggesting that the electronic detection of root canal perforations can be a reproducible technique.
